# The Fanconi anemia associated protein FAAP24 uses two substrate specific binding surfaces for DNA recognition

**DOI:** 10.1093/nar/gkt354

**Published:** 2013-05-09

**Authors:** Hans Wienk, Jack C. Slootweg, Sietske Speerstra, Robert Kaptein, Rolf Boelens, Gert E. Folkers

**Affiliations:** Bijvoet Center For Biomolecular Research, NMR Spectroscopy, Utrecht University, Padualaan 8, 3584 CH Utrecht, The Netherlands

## Abstract

To maintain the integrity of the genome, multiple DNA repair systems exist to repair damaged DNA. Recognition of altered DNA, including bulky adducts, pyrimidine dimers and interstrand crosslinks (ICL), partially depends on proteins containing helix-hairpin-helix (HhH) domains. To understand how ICL is specifically recognized by the Fanconi anemia proteins FANCM and FAAP24, we determined the structure of the HhH domain of FAAP24. Although it resembles other HhH domains, the FAAP24 domain contains a canonical hairpin motif followed by distorted motif. The HhH domain can bind various DNA substrates; using nuclear magnetic resonance titration experiments, we demonstrate that the canonical HhH motif is required for double-stranded DNA (dsDNA) binding, whereas the unstructured N-terminus can interact with single-stranded DNA. Both DNA binding surfaces are used for binding to ICL-like single/double-strand junction-containing DNA substrates. A structural model for FAAP24 bound to dsDNA has been made based on homology with the translesion polymerase iota. Site-directed mutagenesis, sequence conservation and charge distribution support the dsDNA-binding model. Analogous to other HhH domain-containing proteins, we suggest that multiple FAAP24 regions together contribute to binding to single/double-strand junction, which could contribute to specificity in ICL DNA recognition.

## INTRODUCTION

Deficiencies in DNA repair severely limit the life span of cells and result in increased susceptibility to cancer. Patients with Bloom’s syndrome, Xeroderma pigmentosum (XP) and Fanconi anemia (FA) suffer from a variety of severe defects, but all are predisposed to early onset of cancer (1). For these diseases, this is thought to be the result of impaired recombination repair, nucleotide excision repair (NER) and interstrand crosslink (ICL) repair, respectively, owing to mutations in associated repair genes. Although the number of patients suffering from these autosomal recessive genetic disorders is relatively low (∼1:100 000), these diseases play a key role in unravelling the mechanisms underlying the corresponding DNA repair pathways and development of associated cancers. Although inherited cancers are rare, occurring in <10% of all cases, knowledge acquired from these rare hereditary diseases can provide insight into the cause of common sporadic cancers (2).

Fanconi anemia is an inherited disease characterized by genomic instability and hypersensitivity to chemicals, caused by mutations in one of the Fanconi anemia genes leading to lack of interstrand crosslink repair [for reviews see (3–5)]. It is becoming clear that FA proteins contribute to sensing, recognition and processing of ICLs during replication (3–6). In the primary step towards repair, the ICL is recognized by the complex of two proteins, FAAP24 and FANCM. The two proteins both participate in substrate binding and thereby enable recruitment of the FA core complex (7). This FA core complex has E3 ligase activity and can mono-ubiquitinate two other proteins, FANCI and FANCD2. The FANCI/FANCD2 complex recruits other FA repair proteins including FANCJ, FANCN, BRCA1 and BRCA2 (8). Recently, the interaction of FAN1 with ubiquinated FANCD2 was established. Through the recruitment of the FAN1 nuclease to damaged DNA, substrate processing could be performed by its intrinsic 5'-3' exonuclease and endonuclease activity that performs the cleavage of nicked and branched structures (9–12).

The FANCM/FAAP24 complex triggers the initiation of ICL DNA repair by the ability to recruit the FA core complex to chromatin when DNA is damaged (13). Depletion of either protein of the complex causes hypersensitivity to cross-linking agents and chromosomal instability (7); furthermore, FANCM-deficient mice show a FA phenotype (14). Downregulation of either FAAP24 or FANCM does not directly affect integrity of the FA core complex, but the ability to recruit the FA core complex to the cross-linked DNA is lost (13). Depletion of the FAAP24 protein further causes decreased FANCM stability (13) and cell cycle checkpoint response (15), arguing that the two proteins form a heterodimer and act together in DNA binding and recruitment of the FA core complex. This is underscored by the ability of the complex to bind *in vitro* to DNA structures that mimic repair substrates (7). Although Ciccia *et al.* (7) showed that the isolated FANCM C-terminal domain (1727–2048) and full-length FAAP24 can bind either single-stranded DNA (ssDNA) or splayed arm substrates, it was evident that the FANCM/FAAP24 complex binds various DNA substrates significantly better.

**Figure 1.**
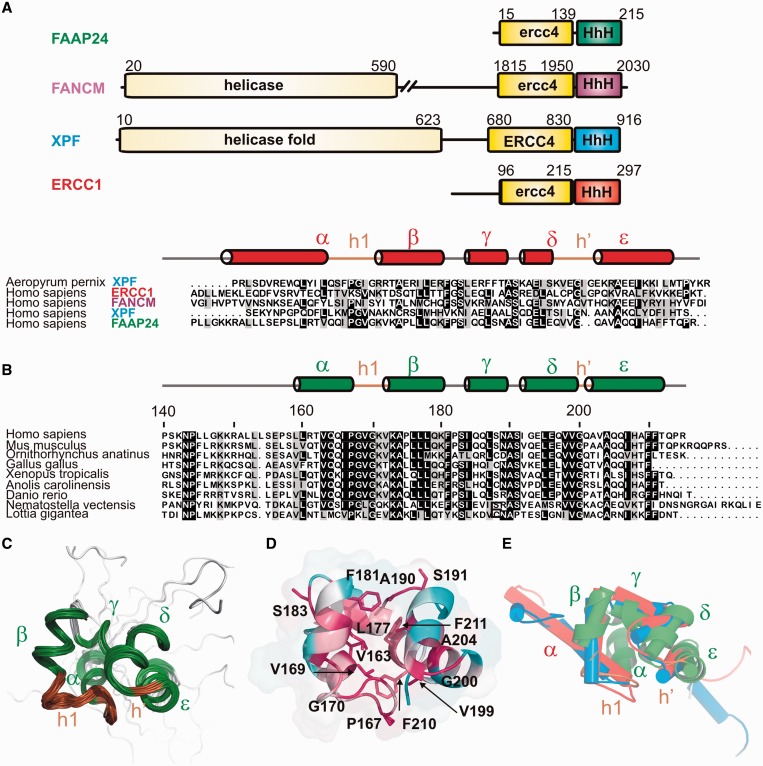
Solution structure of the HhH domain of FAAP24*.* (**A**) Domain organization of FAAP24 (with the HhH domain structure in green), FANCM (purple), XPF (blue) and ERCC1 (red). ERCC4 refers to the catalytically active nuclease domain; ercc4 refers to the structurally homologous domain that lacks endonuclease activity or nuclease signature. The lower panel shows the structure-based alignment of the HhH domain sequences colored using the Boxshade server; for comparison, an archaeal XPF sequence is depicted. The secondary structure elements are indicated for the ERCC1 HhH domain [1z00 (16)], the hairpin regions (h1, h′) are depicted in brown. (**B**) A representative multiple sequence alignment of the FAAP24 protein family (140–215) made using clustalX (17), showing the most distantly related FAAP24 HhH orthologous sequences obtained from the OMA database (18). Coloring is based on all orthologues and performed with Boxshade with a shading threshold at 0.8. Secondary structure elements for FAAP24 HhH domain are depicted above, h’ refers to the second HhH motif that lacks a classical hairpin region. (**C**) Ensemble of the 20 lowest energy structures of the FAAP24 HhH domain (140–215). Secondary structure elements are depicted in green, the hairpin domain regions brown. (**D**) Cartoon representation of the FAAP24 HhH domain structure colored according to sequence conservation, least conserved residues are colored turquoise and most conserved red. The latter residues are depicted in a stick representation. Sequence conservation was calculated using Consurf (19) with the multiple sequence alignment from 1B. The N-terminal tail (140–157) of FAAP24 is structurally disordered and omitted for clarity in most representations. (**E**) Overlay of the HhH domain structures of FAAP24 (green), ERCC1 (red; 1z00), XPF (blue; 1z00) with helices presented as cylinders, the FAAP24 HhH domain structure is also shown in green in cartoon representation.

The domain organization of FANCM and FAAP24 is similar to that of the NER proteins XPF and ERCC1, respectively (Figure 1A). Both contain an ERCC4-like nuclease domain and a C-terminal Helix-hairpin-Helix (HhH) domain (20). HhH domains generally consist of two HhH motifs that together contribute to substrate recognition. Canonical HhH motifs, formed by two parallel folded helices separated by a hairpin motif with a conserved PGφG sequence, bind non-specifically to double-stranded DNA (dsDNA) by making phosphate backbone interactions through the hairpin regions and surrounding positively charged residues (21,22). We and others analyzed the structure and function of these nucleotide excision repair proteins previously and found that the C-terminal HhH domains of XPF and ERCC1 form a stable heterodimer (16,23). The XPF/ERCC1 complex has considerable asymmetry in DNA-binding specificity (16), where ERCC1 binds dsDNA (16) and XPF binds ssDNA (24). The FANCM and FAAP24 proteins show both similarities and differences with XPF and ERCC1. Like XPF, FANCM contains a near consensus nuclease and helicase domain; the domain organization of FAAP24 resembles ERCC1. Both FAAP24 and FANCM contain HhH motifs; however, the sequences deviate significantly from the consensus (Figure 1A). The sequence of the hairpin region of the first HhH motif shows similarity to other HhH family members (21,22), whereas the second HhH motif either completely lacks the consensus sequence (FANCM) or is two residues shorter (FAAP24) (Figure 1A). Based on functions that HhH and ERCC4-like domains fulfil in other XPF family members, we hypothesize that the analogous domains of FANCM and FAAP24 contain distinct DNA-binding domains that together contribute to ICL substrate recognition.

Previously, a model for DNA binding by FANCM/FAAP24 has been proposed based on similarity with ERCC1 and XPF (7). However, experimental structures are lacking, and thus the mechanism underlying substrate preference remains elusive. As a first step to elucidate the mechanism underlying ICL recognition, we determined the solution structure of the HhH domain of FAAP24. We show that the isolated FAAP24 protein is able to bind to both dsDNA and ssDNA using two separate DNA-binding regions. Based on the structural similarity to other HhH domain proteins, the DNA-binding residues, the charge distribution, the hydrophobicity distribution and the sequence conservation, we present a model that can explain the recognition of single/double-strand (ss/ds) junctions in ICL by the FAAP24 HhH domain.

## MATERIALS AND METHODS

### Protein expression and purification

The HhH domain of FAAP24 (139–215) and full-length FAAP24 (1–215) were PCR amplified and cloned into pLICHIS using the previously described enzyme-free cloning strategy (25). FAAP24 mutants were prepared using the QuikChange protocol (Stratagene).

Protein expression and isotopic labeling was performed in the *Escherichia coli* strain BL21 (DE3) Rosetta2 (Novagen) essentially as described before (26). Expression was induced by addition of 0.5 mM IPTG, and cultures were incubated for 16 h at 20°C. Cell pellet was resuspended in 10 ml lysis buffer [50 mM NaHPO_4_ (pH 8.0), 300 mM NaCl, 20 mM imidazole, 1 mM β-mercaptoethanol, 0.2 mM PMSF and 100 μl EDTA-free protease inhibitor cocktail (Sigma)]. After resuspension, 2 mg of lysozyme was added per liter bacterial culture. Following two freeze/thaw cycles and sonication, the sample was cleared by centrifugation for 45 min (17 500g). The N-terminally His-tagged protein was purified on a nickel charged Poros MC20 column (PerSeptive Biosystems). The protein was eluted with elution buffer [50 mM NaHPO_4_ (pH 8.0), 300 mM NaCl, 500 mM imidazole], and following buffer-exchange to gelfiltration buffer [50 mM NaHPO_4_ (pH 8.0), 400 mM NaCl], the protein was applied on a HiLoad 26/60 Sephadex 75 gel-filtration column (GE HealthCare). Purified protein was buffer-exchanged to nuclear magnetic resonance (NMR) buffer [50 mM NaHPO_4_ (pH 6.5), 100 mM NaCl, 8% D_2_O, 0.2 mM PMSF, containing 2 µl of complete protease inhibitor cocktail (Roche)] using a 5 kDa ultrafiltration device (Amicon, Millipore) and concentrated to 200 μM. Owing to the absence of tryptophan or tyrosine residues, quantification and normalization of mutant proteins was performed using SDS–PAGE.

### Electrophoretic mobility shift assay

Electrophoretic mobility shift assay (EMSA) experiments were performed as described before (27) using radiolabeled DNA probes as substrates for FAAP24 binding in either low-salt buffer containing 10 mM Tris (pH 7.0), 10 mM NaCl or high-salt buffer containing 50 mM NaHPO_4_ and 400 mM NaCl. Both buffers further contained 5% glycerol, 1 mM DTT and BSA to a final concentration of 20 µg/ml. All oligonucleotides were purchased from Operon or Eurogentec and annealed when applicable by incubating the two mixed strands (final concentration: 50 μM) in a buffer containing 10 mM Tris (pH 8.0) and 100 mM NaCl for 5 min at 95°C, followed by cooling down for 1 h. After incubation for 30 min on ice, samples were loaded on a 0.5× TBE buffered 5% acrylamide gel, and electrophoresis was carried out for 2.5 h at 140 V at 4°C. Alternatively, samples were loaded on a 0.5× TBE buffered 3.5% agarose gel, and electrophoresis was performed for 1.5 h at 80 V at 4°C. Analysis and quantification was performed as described before (27). The following DNA probes were used: bubble structured DNA (B10) gggcggcgggttttttttttggcggggcgg and ccgccccgccttttttttttcccgccgccc; dsDNA (ds30): gggcggcgggttttttttttggcggggcgg and ccgccccgccaaaaaaaaaacccgccgccc; ssDNA (ss39): tgcgaattcatatgcaatattcagtggctgagctactgg.

### NMR experiments

NMR experiments were recorded at 298 K on Bruker AVANCE 500 and AvanceIII 600 MHz spectrometers with TXI probes, using 0.2 mM [^13^C,^15^N]-labeled FAAP24 HhH domain in NMR buffer. The NMR spectra were processed using Topspin 2.1 (Bruker) or NMRPipe (28) and analyzed with Sparky (T.D. Goddard and D.G. Kneller, University of California, San Francisco). Resonance assignments were obtained using standard 3D triple-resonance experiments. Dihedral angle restraints were obtained from chemical shift information using TALOS+ (29). Distance restraints for structure calculations were obtained from 2D [1H,^1^H]-NOESY (mixing time 120 ms), 3D NOESY-[^1^H,^15^N]-HSQC (mixing time 100 ms) and 3D NOESY-[^1^H,^13^C]-HSQC spectra (mixing time 120 ms). For NMR dynamics, conventional ^15^N relaxation measurements were performed (T_1_, T_2_ and ^15^N-NOE) and analyzed using a model-free approach yielding S^2^ order parameters (30).

For NMR titrations of FAAP24, the following substrates were used: dsDNA (ds10): gggcggcggg and cccgccgccc; (ds20): ggcggggcgggggcggcggg and cccgccgcccccgccccgcc ssDNA, (ss20): tttttttttttttttttttt; a DNA bubble structure (B10): gggcggcgggttttttttttggcggggcgg and ccgccccgccttttttttttcccgccgccc and a splayed arm (F10): gggcggcgggtttttttttt and ttttttttttcccgccgccc. For splayed arm titration experiments with 3′ and 5′ extended ssDNA, the oligo’s GATTCTAAAGTTAGATAGGCcccgccgccc and gggcggcgggCGGATAGATTGAAATCTTAG were annealed, whereas for the 5′ extension, the first oligo was annealed with gggcggcggg; for the 3′ extension, the second oligo was annealed with cccgccgccc. DNA was annealed in NMR buffer to a final concentration of 1.25 or 2.5 mM. A series of [^15^N,^1^H]-HSQC spectra were acquired with the successive addition of DNA substrates (12.5–200 μM) to 50–100 μM FAAP24 HhH domain or full-length FAAP24 protein samples. The data were processed and analyzed as described before (27).

### Structure calculation

Automated NOE assignment and structure calculations were performed essentially as described before (31) using CYANA 2.2 (32). Water refinement was performed with CNS (33) and validated according to the RECOORD protocol (34). The structures were analyzed using WHATCHECK (35) and PROCHECK (36).

NMR chemical shift assignments (140–215) were deposited in the Biological Magnetic Resonance Bank database with entry code 18725. Coordinates for the 20 lowest energy structures were deposited in the Protein Data Bank under accession code 2lyh.

## RESULTS

### Solution structure of the FAAP24 HhH domain

Recently, we found that the second HhH motif of the human repair protein XPF has a non-canonical hairpin sequence in which one residue is missing resulting in a distorted structure that permits specific recognition of ssDNA (24). Amino acid sequence comparison indicated that FAAP24 lacks two residues in the second hairpin motif (Figure 1A). Therefore, a substantial structural deviation and a different substrate preference compared with canonical HhH domains is expected.

Based on sequence comparison and structure prediction, we determined the C-terminal boundary of the ERCC4-like domain of FAAP24 (1–139) and expressed the HhH domain (139–215) referred to as FAAP24 HhH domain. Gelfiltration profile, NMR diffusion data and T1/T2 relaxation experiments indicated that the FAAP24 HhH domain is monomeric in solution (data not shown). The structure was calculated using 1172 distance restraints and 120 dihedral angle restraints; a summary of all structural and restraints statistics is given in Table 1. The calculations show that the N-terminal region (140–157) connecting the ERCC4-like domain and the HhH domain show considerable disorder (Figure 1C). This is supported by chemical shift analysis using the program CSI (37) that did not reveal the presence of regular secondary structure elements. Although TALOS+ analysis did suggest the presence of short extended structures, it predicted reduced generalized order parameters (S^2^) for this FAAP24 linker. Also, heteronuclear ^15^N{^1^H}NOE experiments indicate that the N-terminal region is flexible (data not shown). For the well-defined parts (158–215) of the 20 lowest energy structures, the average RMSD to the mean coordinates are 1.01 and 0.56 Å for all heavy and the backbone atoms, respectively.

**Table 1.** Statistics for the FAAP24 HhH domain structure ensemble

**Number of NOE crosspeaks**			
	*2D HH-NOE*	*3D HNH NOE*	*3D HCH-NOE*
Total	2279	271	3513
Assigned	1711	692	844
**Number of NOE restraints in the final structure calculation**			
Intraresidual/sequential		695	
Medium-range^a^		326	
Long-range		151	
	
Total		1172	
**Other restraints**			
TALOS dihedral angle restraints		120	
**Violations**			
NOE distances violations >0.5 Å		0	
Dihedral angle violations >5°		0	
**RMS deviation from mean (Å)**			
All heavy atoms		1.01	
Backbone atoms (N, C^α^, C')		0.56	
**Ramachandran plot statistics^b^**			
Most favored region (%)		95.6	
Additionally allowed region (%)		4.2	
Generously allowed region (%)		0.2	
Disallowed region (%)		0.0	
**WHAT CHECK structure *Z*-scores^b^**			
Ramachandran plot appearance		−2.2	
Second generation packing quality		−2.4	
χ^1 ^− χ^2^ rotamer normality		−0.9	
Backbone conformation		−2.1	

^a^1 < |i − j| < 5.

^b^Values are for the core of the protein, i.e. S^158^−R^215^.

FAAP24 adopts an HhH domain-like fold consisting of five α-helices (Figure 1). Helices α and β form a well-defined hairpin-like motif, but the orientation of helix α is distinct from other HhH family members, as this helix packs against helix ε involving van der Waals contacts between V163, I166, I207, F210 and F211. The second (pseudo) HhH motif lacks a typical hairpin sequence. Instead, a turn is formed around the fully conserved G200 that separates the helices δ and ε (Figure 1D). Thus, despite the absence of the hairpin sequence, the geometry of the second HhH motif is well preserved, involving van der Waals contacts between the flanking highly conserved hydrophobic residues L195 and A204. Owing to these structural differences, the helical angles between the various helices deviate from the average HhH domain structure. Furthermore, the distance between the two hairpin motifs is shorter than for other HhH domain structures (Figure 1E).

### The HhH domain of FAAP24 binds DNA

Despite the structural differences aforementioned, analysis of the structure of the well-defined part of the FAAP24 HhH domain (residues 159–215) using the Dali (38) revealed significant similarity with other HhH domain-containing proteins. The largest structural similarity was found with Pyrococcus furiosus XPF (39) (Supplementary Figure S1), with a *Z*-score of 5.7 and a backbone RMSD of ∼ 2.0 Å. High similarity was also observed with several other HhH domain protein structures that were determined in the presence of DNA (Supplementary Figure S1). The largest homology (*Z*-score 4.7) to a nucleic acid-bound protein was obtained with the HhH domain of homodimeric XPF bound to ssDNA (24). Limited homology (*Z-*score 2.1) was further found with the HhH domains present in RuvA bound to Holliday junction DNA (data not shown) (40). Finally, significant structural similarity (*Z*-score 3.7) was detected for the HhH-like motif present within various DNA polymerases (41,42).

**Figure 2.**
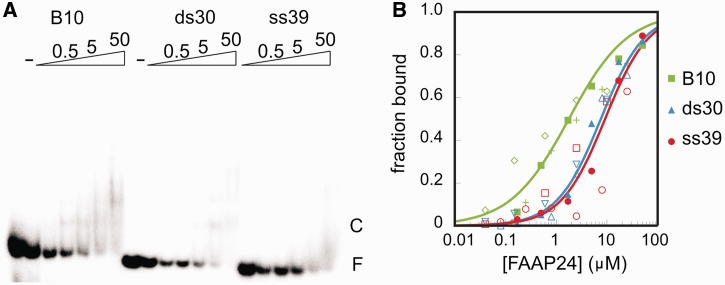
Nucleic acid binding of the HhH domain of FAAP24. (**A**) Representative EMSA with a 39 nucleotides ssDNA (ss39), a 30 bp dsDNA (ds30) and a 30 bp bubble DNA (B10) consisting of two 10 bp dsDNA stems separated by 10 unpaired nucleotides. This binding assay is performed in the presence of 0 (−), 0.2, 0.5, 1.7, 5, 16.7, 50 μM FAAP24 HhH domain protein. The complex is marked as ‘C’, the free DNA as ‘F’. (**B**) Fraction bound DNA is plotted as a function of the FAAP24 HhH domain protein concentration. Experiments were performed in triplicate using B10 (green), ds30 (blue) ss39 (red); for each individual binding experiment, a different symbol is used, and the line represents the calculated binding curve based on the quantified apparent dissociation constant based on all data points, R^2^ of 0.9, 0.98, 0.92 for B10, ds30 and ss39 respectively.

Structural homology with DNA-bound HhH motif-containing proteins supports the idea that also the HhH domain of FAAP24 binds DNA. To test this, we performed EMSA using dsDNA, ssDNA or the B10 probe containing an ss/dsDNA junction (Figure 2). Although disappearance of free DNA was detectable, formation of the protein–DNA complex was not proportional to the disappearance of free DNA, suggesting that the protein dissociates during electrophoresis. The binding affinities of the FAAP24 HhH domain for ssDNA and dsDNA are similar, with an apparent K_d_ of 7.8 ± 0.4 µM and 9.1 ± 0.9 µM, respectively. The affinity for B10 DNA was significantly higher with an apparent K_d_ of 1.2 ± 0.2 µM, although it should be mentioned that multiple complexes are formed, in a non-cooperative fashion. This can be explained by the presence of two ss/dsDNA junctions. Dissociation during electrophoresis and formation of multiple comlexes complicates calculation of the apparent dissociation constants. Other methods are presently evaluated to determine binding affinities for various probes. Using a high-percentage agarose gel instead of a polyacrylamide gel, the B10-FAAP24 complex could be stabilized (Supplementary Figure S2), whereas for the other two probes, dissociation during electrophoresis remained. The binding appears, at least partially, electrostatic in nature. This is supported by the finding that binding in the presence of 400 mM instead of 10 mM NaCl led to a 3–4-fold decrease in affinity for B10 DNA, whereas binding to other substrates became too weak to quantify reliably (data not shown). Furthermore NMR titration experiments in the presence of 400 mM NaCl, instead of 150 mM NaCl, using a 20 bp dsDNA fragment or a 30 nt ssDNA revealed a significantly weaker binding, evidenced by much smaller chemical shift changes on addition of nucleic acids (Supplementary Figure S6). Similarly, ∼10–20% of the maximal chemical shifts obtained in the presence of 100 mM NaCl were obtained in the presence of 400 mM NaCl for the splayed arm substrate (Supplementary Figures S4–S6). The small chemical shifts with 400 mM NaCl at 25°C in the presence of a 2-fold excess of DNA is indicative for at least an order of magnitude weaker binding, suggesting that at higher temperature, the interaction is more affected by the addition of ions. Taken together, these results underscore the ability of the FAAP24 to bind various DNA substrates and show that the binding affinity is at least partially electrostatic in nature.

### DNA binding surface characterized by NMR

**Figure 3.**
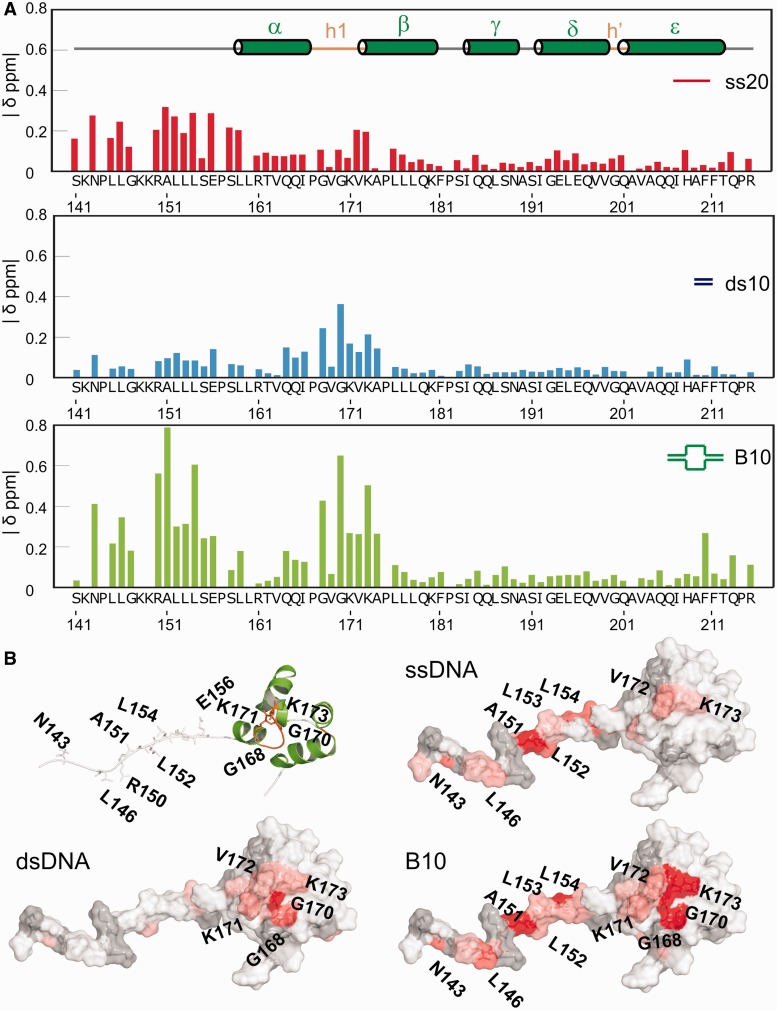
The ssDNA and dsDNA interaction surfaces of the FAAP24 HhH domain. (**A**) NMR CSPs of 100 μM FAAP24 HhH domain protein with different DNA substrates. Compound CSP values were calculated as described before (16) and correspond to the addition of 150 μM ssDNA (ss20, red), 200 μM of dsDNA (ds10, blue) or 150 μM bubble DNA (B10, green). Secondary structure elements and hairpin regions are depicted in the top panel. (**B**) Surface representation of the FAAP24 HhH domain with CSPs on addition of the various DNA substrates plotted on the surface from white [composite CSP (ppm) <0.10; <0.10; <0.15 for ss20, ds10 and B10, respectively] to red (>0.3; >0.3: >0.4 for ss20, ds10 and B10, respectively). Gray indicates residues that could not be assigned or signals that disappeared in the titration.

The binding of FAAP24 to DNA fragments with micromolar affinity permits the identification of the interacting residues using NMR chemical shift perturbations (CSP) in titrations using various DNA substrates. These experiments show that the overall fold of the FAAP24 HhH domain remains unaltered, as only localized spectral changes were observed on addition of ssDNA, dsDNA, splayed arm and B10 (Figure 3 and Supplementary Figures S3–S5). For most peaks, fast exchange is found, although for few residues intermediate exchange is detected, as these peaks show broadening (Supplementary Figures S4 and S5). These exchange regimes on the NMR timescale are indicative for micromolar affinities, in good agreement with EMSA results described earlier in the text. These experiments indicate that, despite the different conditions used for EMSA or NMR experiments (see ‘Materials and Methods’ section), both conditions permit, equally effectieve complex formation. The binding to dsDNA was largely restricted to the hairpin 1 region, where especially backbone amide residues around the positively charged residues within helix β show chemical shift changes. The binding of FAAP24 HhH domain to ssDNA resulted in CSPs in the unstructured N-terminal region of the protein. The binding to the ss/dsDNA probe (B10) led to chemical shift changes for amide resonances in both the N-terminal region and HhH motif 1. We further analyzed the binding of FAAP24 HhH domain to a splayed arm, a substrate resembling the DNA structure formed at stalled replication forks. Chemical shift changes on addition of the splayed arm substrate resembled the changes for the B10 probe, but for the first hairpin motif region, significantly larger shifts were obtained. Interestingly, a probe containing a 5′extension gave chemical shift changes comparable with the splayed arm substrate, whereas the probe with a 3′extension was giving smaller chemical shifts changes (Supplementary Figures S4 and S5). These results are indicative for lower affinity and possibly a different binding mode for the probe containing a 3′ssDNA overhang. These results argue that the DNA-binding domains together could contribute to the polarity of substrate binding. Figure 3B shows the CSPs for the three DNA fragments on the surface of the FAAP24 HhH domain structure. The figure underscores that binding largely leads to localized shifts for ssDNA and dsDNA, and that for B10 DNA and the splayed arm DNA (Supplementary Figures S4 and S5), both binding regions are affected. The complementary contributions of the single-strand and double-strand parts to the DNA binding of FAAP24 HhH domain was further established by the presence of similar chemical shift changes on addition of B10 DNA (Figure 3) or a splayed arm containing one or two ssDNA strands (Supplementary Figures S4 and S5). In contrast, at the high salt conditions, insignificant CSP were obtained using either ssDNA or the splayed arm substrate for the linker between the ERCC4 and HhH domains (Supplementary Figure S6). However, we do find significant changes under these conditions in the dsDNA-binding region using both dsDNA and splayed arm substrates (Supplementary Figure S6). This indicates that especially the ssDNA binding of FAAP24 is influenced by the addition of salt.

Titration experiments of full-length ^15^N-labeled FAAP24 with a 10 bp dsDNA probe show that also in the full-length protein, the first hairpin region is affected (Supplementary Figure S7). Additional CSPs can be seen in the extreme C-terminus of FAAP24. Similar, albeit less pronounced, changes were found for the FAAP24 HhH domain in isolation, using B10 (Figure 3) and a splayed arm substrate (Supplementary Figure S5), but not with dsDNA. These experiments with full-length FAAP24 further reveal that the ERCC4-like domain is affected by the addition of dsDNA. As resonance assignments for this domain are lacking, it is unknown which residues interact with dsDNA.

### DNA binding characterized by mutagenesis

The importance of the HhH motif 1 for DNA binding was verified using mutagenesis. Mutations were introduced in the FAAP24 HhH domain and analyzed by *in vitro* binding assays (Supplementary Figure S2). The combined mutation of residues the K171 and K173 to alanine in helix β led to a 4-fold decrease in binding affinity, whereas the affinity of the individual K173A mutant was 3-fold reduced. The mutations R161A and V162A in helix α led to 2-fold lower binding affinities. As controls, Q205 and Q206, both present in helix ε of the second HhH motif, could be mutated simultaneously to alanines without significant effect on the affinity of FAAP24 HhH domain to B10 DNA.

The combined *in vitro* DNA-binding results and NMR titration data clearly show that the linker region between ERCC4 and the HhH domain and the first HhH motif, but not the distorted HhH motif 2 of FAAP24, are involved in DNA binding.

## DISCUSSION

Despite the absence of a canonical second hairpin region, the solution structure of the HhH domain of FAAP24 shows an overall fold that is similar to other HhH domains. *In vitro* DNA-binding assays demonstrate micromolar affinity of the FAAP24 HhH domain for various nucleic acids. Different from other HhH domain proteins, NMR experiments revealed the presence of two separate DNA-binding regions for FAAP24. One is present in the region corresponding to the linker between the ERCC4-like domain and the HhH domain and the other in the first HhH motif, recognizing ssDNA and dsDNA, respectively.

### Structure

Although experiments indicated that the HhH domain of FAAP24 is monomeric in solution, earlier work demonstrated that for full activity, heterodimerization with FANCM is required (7). In all dimeric HhH domain structures studied, helices α and γ pack against the corresponding helices of the dimer partner. For FAAP24, the position of helix α is not compatible with such a model, as helix α packs against helix ε, thereby possibly preventing dimer formation. It could be that the heterodimeric structure of FANCM/FAAP24 is distinct from other HhH proteins; however, it is more likely that the orientation of the first helix of the HhH domain will rearrange on heterodimerization with FANCM. Owing to poor expression and solubility of the FANCM/FAAP24 heterodimer, we were, so far, unable to structurally analyze this complex.

The structural similarity among HhH domains of members of the XPF family is generally high (*Z-*score of 7.7–10.5) (44). However, similarity of FAAP24 HhH domain to XPF family members is significantly lower (*Z*-score of 3.7–5.5). Interestingly, despite the more similar domain organization of FAAP24 to ERCC1, the HhH domain structure shows more similarity to the human XPF than to the ERCC1 HhH domain structure (16,23) (Figure 1 and Supplementary Figure S1). We have previously shown that XPF has a wider groove between the hairpin motifs 1 and 2 that binds a guanine nucleotide (24). Here, we find a shorter distance between the two hairpin motifs for FAAP24 (8 Å), whereas ERCC1 and XPF have widths of 9 Å and 10 Å, respectively. We propose that the loss of the hairpin residues led to a difference in the HhH domain structure, which in turn led to a distinct substrate binding preference and mechanism. Although the second hairpin motif of ERCC1 is important for dsDNA binding (16), both for XPF and FAAP24, this part is not important. It is tempting to speculate that variations in the second hairpin motif are crucial for specificity in DNA recognition.

**Figure 4.**
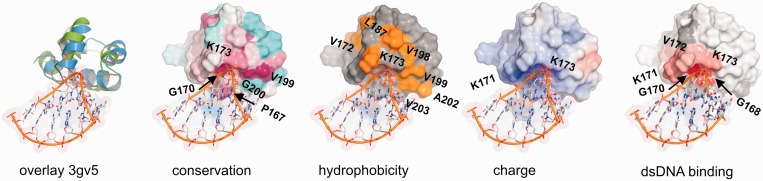
Model for binding of FAAP24 HhH domain to dsDNA. From left to right, following figures are presented: model of the FAAP24 HhH domain bound to dsDNA, based on structural similarity (*Z-*score: 3.7) of FAAP24 HhH domain (green) with DNA polymerase ι [3gv5; (42)]. (blue). Surface representation indicating sequence conservation calculated as in Figure 1D. For indicating hydrophobicity, side chains of hydrophobic residues are colored in orange. For charge, the surface is colored according to the electrostatic surface potential, calculated using APBS (43) (blue: positive, red: negative). The right panel shows determined dsDNA-binding surface colored as described in Figure 3.

The second HhH motif of FAAP24 lacks a canonical hairpin motif but instead contains a turn formed between the two helices around a highly conserved G200. The orientation of the δ and ε helices of FAAP24 shows similarity to the two helices following the canonical HhH motif of DNA polymerases (Supplementary Figure S1) (21). Like FAAP24 HhH domain, the DNA polymerase family Y members revI, pol κ, pol ι [for a review see (45)] contain a canonical HhH motif but lack the second hairpin motif. Instead, the last two helices are separated by a conserved small residue, thereby forming a tight turn with a similar topology as the FAAP24 HhH domain (Figure 4 and Supplementary Figure S1). This structural homology with one of the domains involved in non-specific DNA binding of DNA polymerases agrees well with a role in nucleic acid binding for the HhH domain of FAAP24.

### dsDNA binding

Like other HhH domain proteins, the HhH domain of FAAP24 can bind dsDNA with micromolar affinity (Figure 2). This apparent dissociation constant differs with the findings of Ciccia *et al*. (7), who reported that FAAP24 failed to bind dsDNA. We assume that differences in experimental conditions, such as domain boundaries of the used FAAP24 protein (HhH domain versus full-length protein), protein concentrations, dsDNA sequences and salt concentrations, can explain the differences between the two findings. The work of Ciccia further revealed that both ssDNA and a splayed arm would be a good substrate. We performed NMR titrations (Figure 3 and Supplementary Figures S4 and S5) and EMSA experiments (Figure 2; data not shown), showing that FAAP24 HhH domain binds these substrates, although somewhat less effectively than the B10 probe, a substrate containing two ss/ds-junctions used here.

In almost all HhH motif-containing structures, including DNA polymerases, DNA contacts are found involving both the first HhH motif and the second (distorted) HhH motif (helix δ and ε) (46). The HhH-like fold, located within the thumb domain of the Y-family of DNA polymerases, generally makes contacts with both the phosphoribose backbone of the primer and of the template strand. As for FAAP24 we did not find any chemical shift changes for the second distorted HhH motif, we conclude that FAAP24 has a different binding mode. Interestingly for polymerase ι, involved in translesion synthesis (47), only the canonical HhH motif interacts with the primer strand, and no contacts between the template strand and the non-canonical HhH motif formed by helices δ and ε were observed. Although the structural homology of FAAP24 to this polymerase is somewhat lower than to other XPF family members, the HhH domain of polymerase ι was among the most homologous non-XPF family members (Supplementary Figure S1). This structural homology to the DNA polymerases was used to model dsDNA to the first HhH motif of FAAP24 (Figure 4). The plausibility of the model is supported by the relatively high sequence conservation of the first hairpin region of FAAP24, including the dsDNA interface arginine and lysine residues, of which one (K173), is found in all species (Figure 1B). Indeed, mutation of this residue to alanine led to a 3-fold decrease in binding affinity (Supplementary Figure S2). The presence of a positive surface patch around the dsDNA-binding interface, as found by calculating the electrostatic surface potential (43), supports the proposed model (Figure 4). Furthermore, a small number of surface exposed hydrophobic residues are found at the proposed protein–DNA interface that might make additional DNA contacts (Figure 4).

### ssDNA binding

A remarkable difference between the HhH domain of FAAP24 and the HhH domain of other proteins is the ability of FAAP24 to bind both ssDNA and dsDNA with similar affinity (apparent K_d_ ∼ 10 μM). Surprisingly, on addition of ssDNA, the most pronounced spectral changes were found in the N-terminal unstructured region preceding the FAAP24 HhH domain, corresponding to the linker between ERCC4 and the HhH domain (Figure 3). We found, based on the observed chemical shift changes, no proof for secondary structure element formation on binding to ssDNA (data not shown). This ssDNA-binding region is distinct from the previously characterized ssDNA-binding site of XPF (24). The ssDNA binding appears mainly electrostatic in nature, as it is strongly reduced by increasing salt concentration (Supplementary Figure S6). A possible explanation for the electrostatic effect can be direct interactions of the positively charged residues in the N-terminus of FAAP24 with the negatively charged phosphate backbone of ssDNA.

### Role of FAAP24 in ICL recognition

Based on the structural resemblance of the HhH domain structure of FAAP24 with DNA-bound polymerases, we propose a model for the recognition of ICL-like DNA structures. We argue that the HhH domain of FAAP24 could recognize ss/dsDNA junctions at stalled replication forks formed by the presence of an ICL. The first HhH motif would bind dsDNA, and the linker between the ERCC4-like domain and the HhH domain would interact to ssDNA, thereby providing the specificity for ss/dsDNA junction structures. The binding affinity of the FAAP24 HhH domain for various DNA substrates is at least an order of magnitude lower than for the heterodimeric ERCC4 and HhH domain-containing FANCM/FAAP24 complex (7). In analogy, with other DNA repair proteins including ERCC1/XPF (20), we postulate that also the HhH domain of FANCM and the ERCC4-like domains of both proteins may contribute to substrate binding. Indeed, NMR titrations support an additional role for the ERCC4-like domain of FAAP24 in dsDNA binding (Supplementary Figure S7). With the heterodimeric FANCM/FAAP24 complex, the individual DNA-binding domains could act together, thereby providing both affinity and specificity. Further structural studies involving all DNA-binding domains of FANCM/FAAP24 are required to elucidate how the FANCM/FAAP24 heterodimer specifically recognizes ICL-like structures.

## ACCESSION NUMBERS

BMRB: 18725, PDB: 2lyh.

## SUPPLEMENTARY DATA

Supplementary Data are available at NAR Online: Supplementary Figures 1–7.

## FUNDING

EU programs Structural Proteomics in Europe (SPINE) [QLG2-CT-2002-00988]; SPINE2-complexes [Contract 031220]; EU-NMR [Contract RII3-026145]; BioNMR [Contract 261863]; and the Division of Chemical Sciences of the Netherlands Organization for Scientific Research (NWO-CW). Funding for open access charge: Utrecht University (in part).

*Conflict of interest statement.* None declared.

## Supplementary Material

Supplementary Data
