# Supplementary material for: The Fanconi anemia associated protein FAAP24 uses two substrate specific binding surfaces for DNA recognition
Source: Nucleic Acids Res. 2013 May 9;41(13):6739–49. doi: 10.1093/nar/gkt354 (PMC3711432; doi:10.1093/nar/gkt354)
Supplement: Supplementary Data [file supp_41_13_6739__index.html]

The Fanconi anemia associated protein FAAP24 uses two substrate specific binding surfaces for DNA recognition — The Fanconi anemia associated protein FAAP24 uses two substrate specific binding surfaces for DNA recognition — Supplementary Data 

# The Fanconi anemia associated protein FAAP24 uses two substrate specific binding surfaces for DNA recognition

## Supplementary Data

files

**Files in this Data Supplement:**

- Supplementary Data - doc file
