# Supplementary material for: The Fanconi anemia associated protein FAAP24 uses two substrate specific binding surfaces for DNA recognition
Source: Nucleic Acids Res. 2013 May 9;41(13):6739–49. doi: 10.1093/nar/gkt354 (PMC3711432; doi:10.1093/nar/gkt354)
Supplement: Supplementary Data [file supp_gkt354_nar-00042-m-2013-File006.doc]

**Supplementary material.**

Article: The Fanconi Anemia associated protein FAAP24 uses two substrate specific binding surfaces for DNA recognition

Authors: Hans Wienk, Jack C. Slootweg, Robert Kaptein, Rolf Boelens and Gert E. Folkers


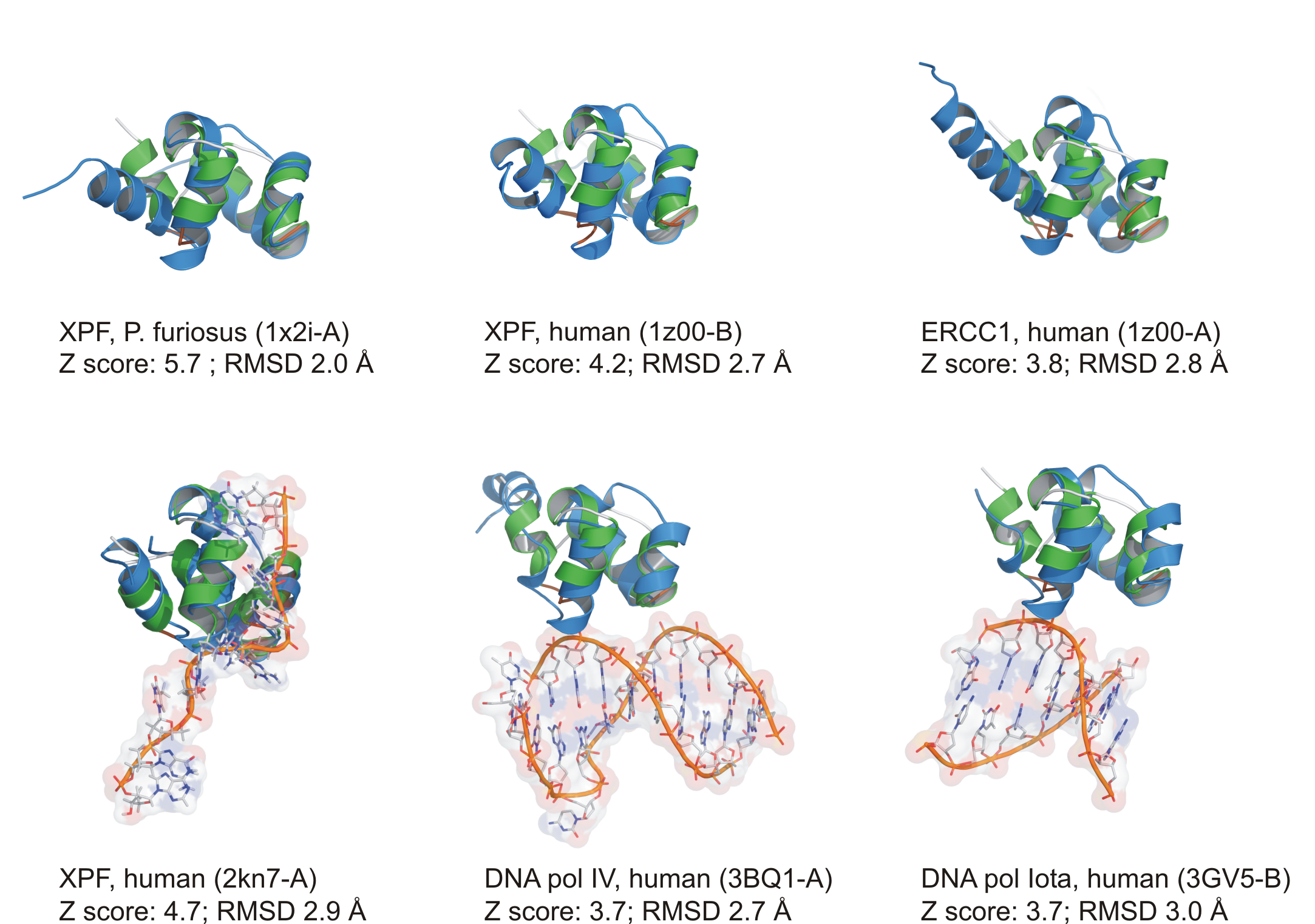


**Supplementary Figure S1.** *Structural similarity of the HhH domain of FAAP24 with other HhH domain proteins and DNA polymerases.*

The data are obtained using the Dali server (34) with the structure of the HhH domain of FAAP24 (158-215) as input. The structure of FAAP24 HhH in green and various structural homologs in blue. The name of the protein, the organism, the PDB entry code, the Z score and root mean square deviation between the two structures is given for six representative structural homologs.

**
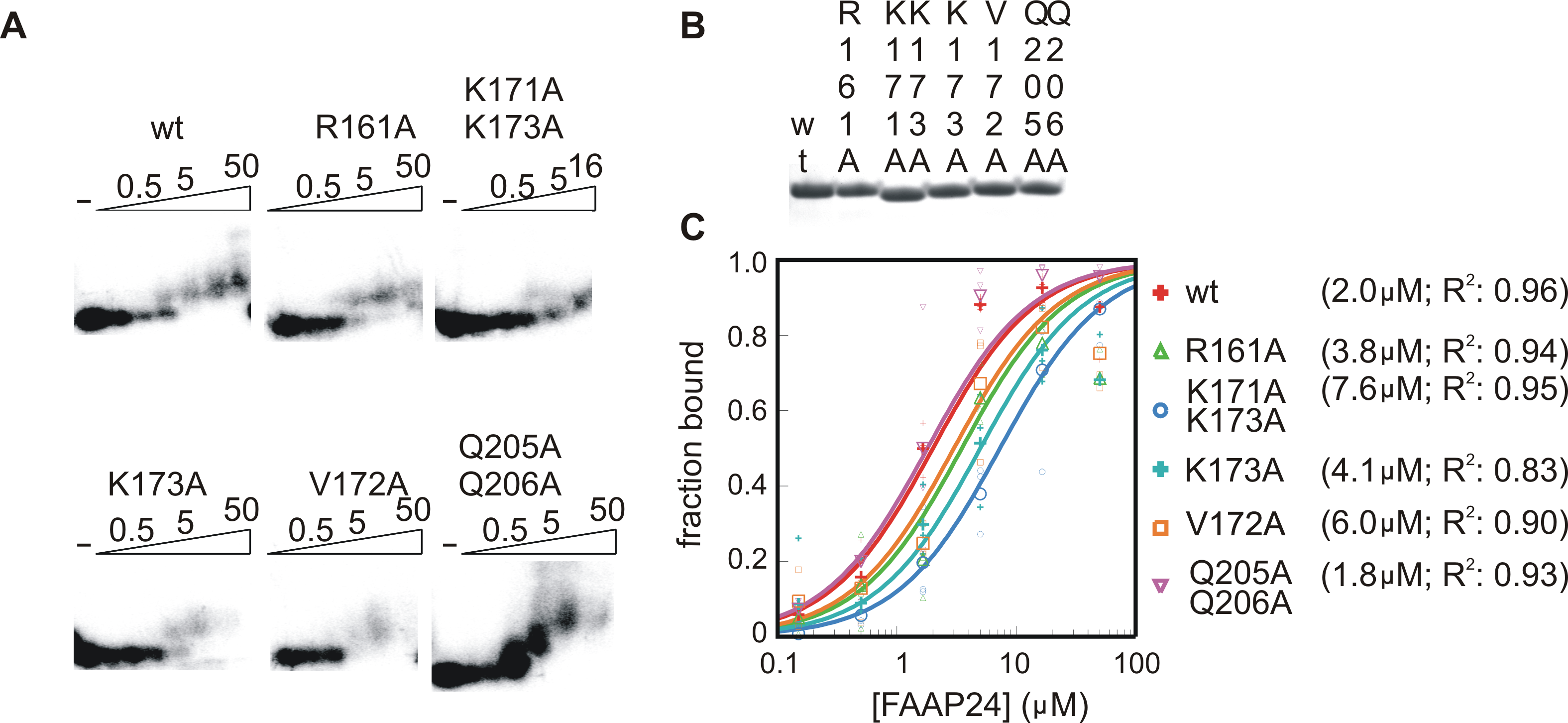
**

**Supplementary Figure S2.** *Effect of mutations in HhH motif 1 on the DNA binding of FAAP24.*

A) EMSA DNA binding assays using B10 DNA as a probe, in the presence of 0 (-), 0.2, 0.5, 1.7, 5, 16.7, 50 μM of FAAP24 HhH domain wild type (wt) and different mutant proteins. B) SDS page showing the proteins used for DNA binding experiments. C) Quantification of EMSA experiments. The fraction bound DNA is plotted as a function of the FAAP24 HhH domain protein concentration. The average fraction bound of three independent experiments is indicated in large symbols, the small symbols refer to the individual experiments. The line shows the binding curve using the calculated apparent K_d_;; this value and the R^2^ of the fit is given for each mutant.


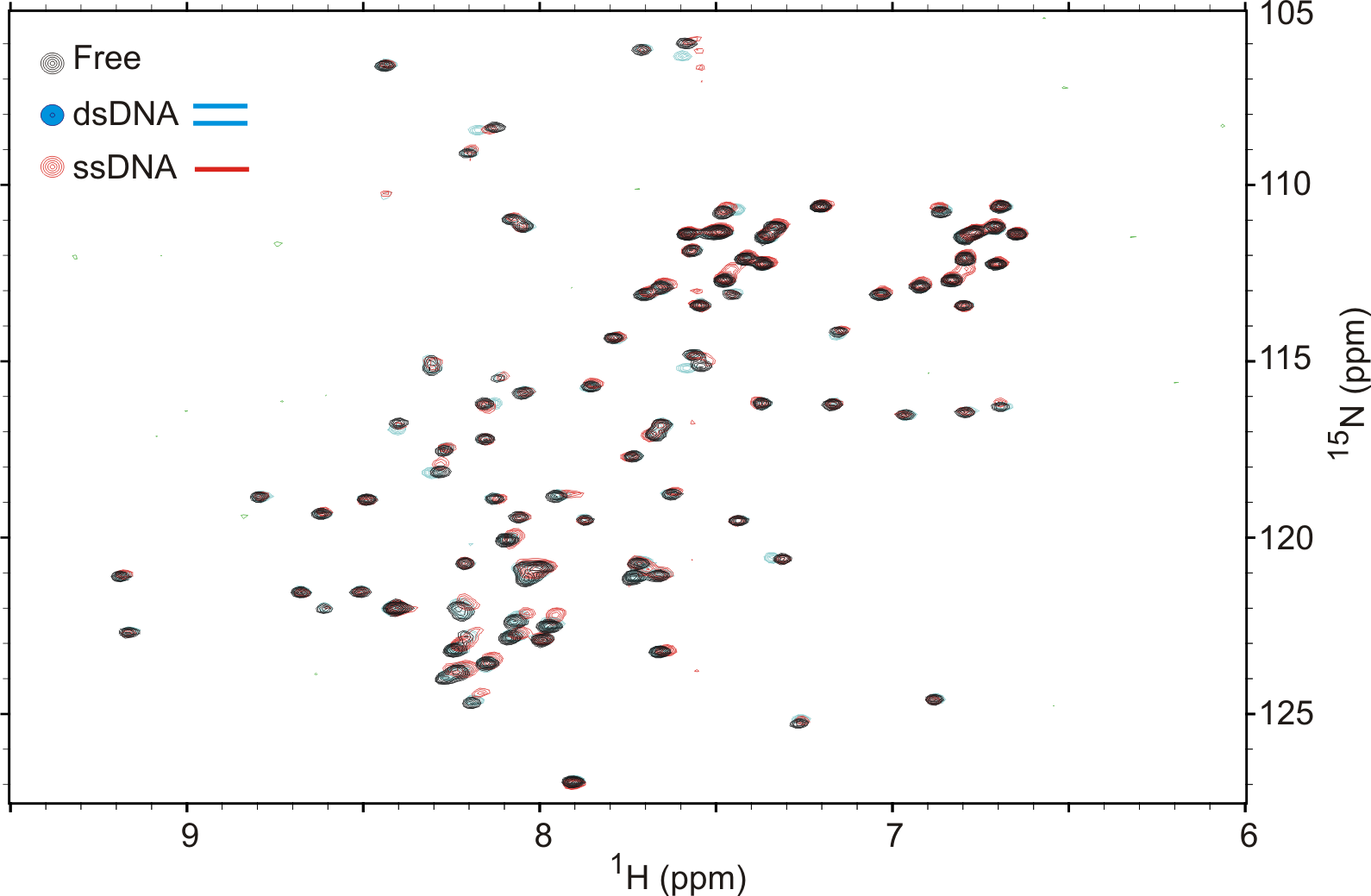


**Supplementary Figure S3*.*** *[^15^N,^1^H]-HSQC spectrum of 100 μM FAAP24 HhH domain in the presence of ssDNA or dsDNA*

Shown are the spectra of 100 μM of FAAP24 HhH domain in the absence (black) or presence of 150 μM ssDNA (ss20, red) or 200 μM dsDNA (ds10, cyan).


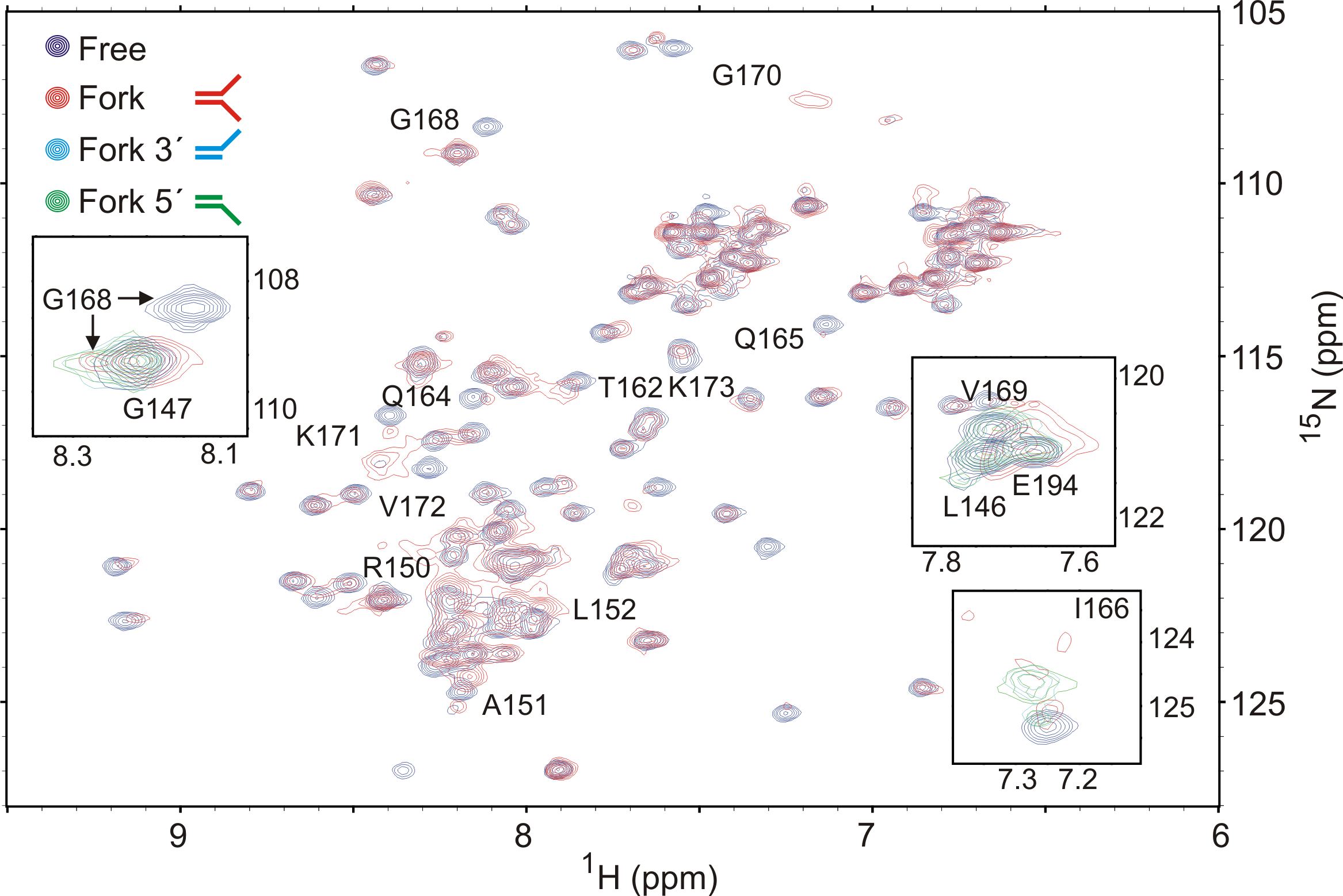


**Supplement Figure S4** *^15^N HSQC spectrum of the HhH domain of FAAP24 in the presence of splayed arm substrates*

^15^N HSQC spectrum of 50 μM of Faap24 HhH domain in the absence (blue) or presence of 100 μM splayed arm substrate (Fork, 10 basepairs and 20 nucleotides ssDNA (red). The inset show few affected residues for a splayed arm (red), a fork with 3´extension (cyan) or 5´extension (green).

**
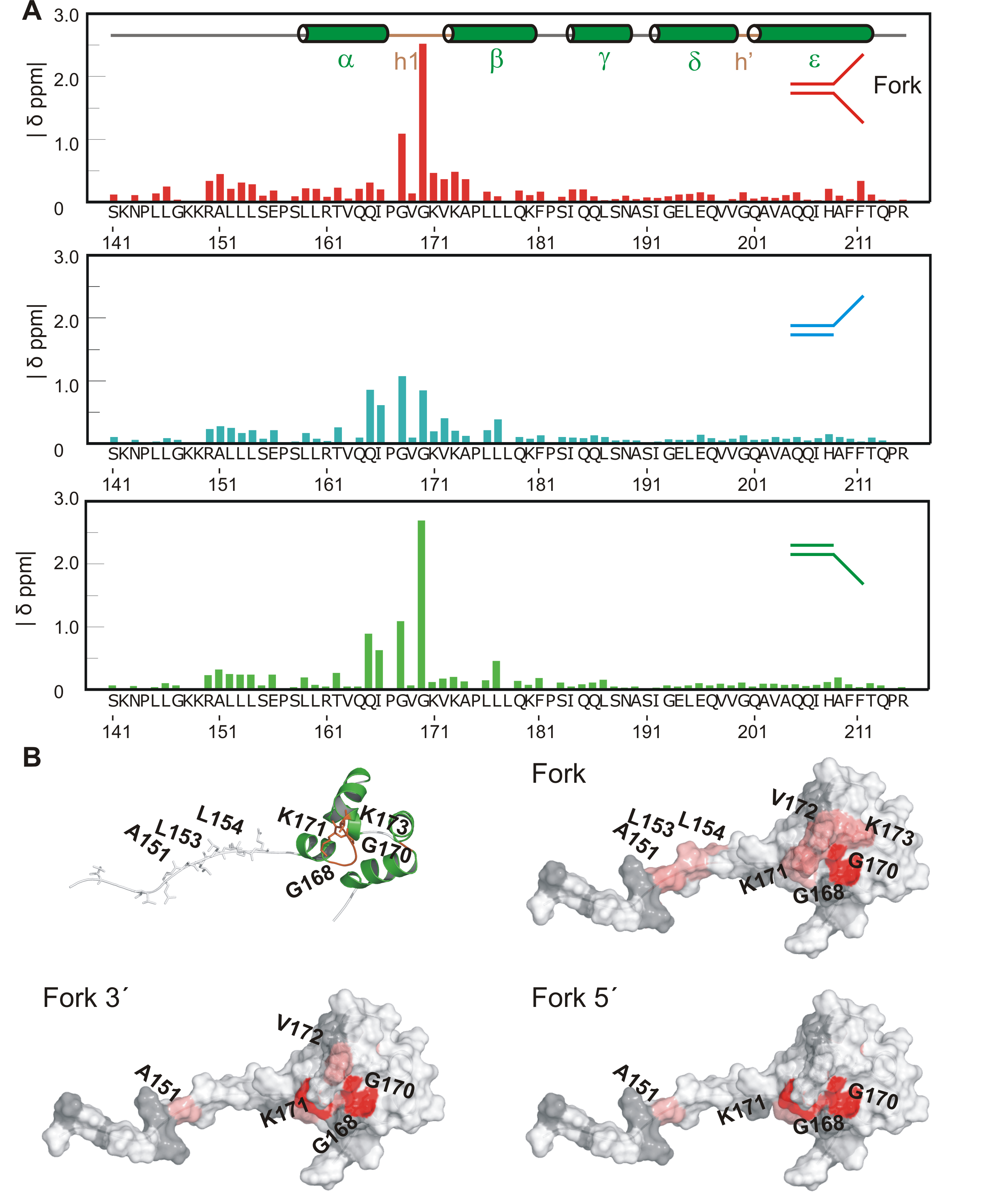

Supplement Figure S5** *The interaction surfaces of the FAAP24 HhH domain for a splayed arm probe.*

A) NMR CSPs of 50 μM FAAP24 HhH domain protein with splayed arm DNA substrates. Compound CSP values were calculated as described in Figure 3, and correspond to the addition of a two fold excess of Splayed arm (5´and 3´extension) (Fork, red), Splayed arm with a 3´ss DNA extension (cyan) or splayed arm with a 5´ssDNA extension splayed arm DNA (green). Secondary structure elements and hairpin regions are depicted in the top panel. B) Surface representation of the FAAP24 HhH domain with CSPs upon addition of the various DNA substrates plotted on the surface from white (composite CSP (ppm) <0.25 to red >1.0). Gray indicates residues that could not be assigned or signals that disappeared in the titration.


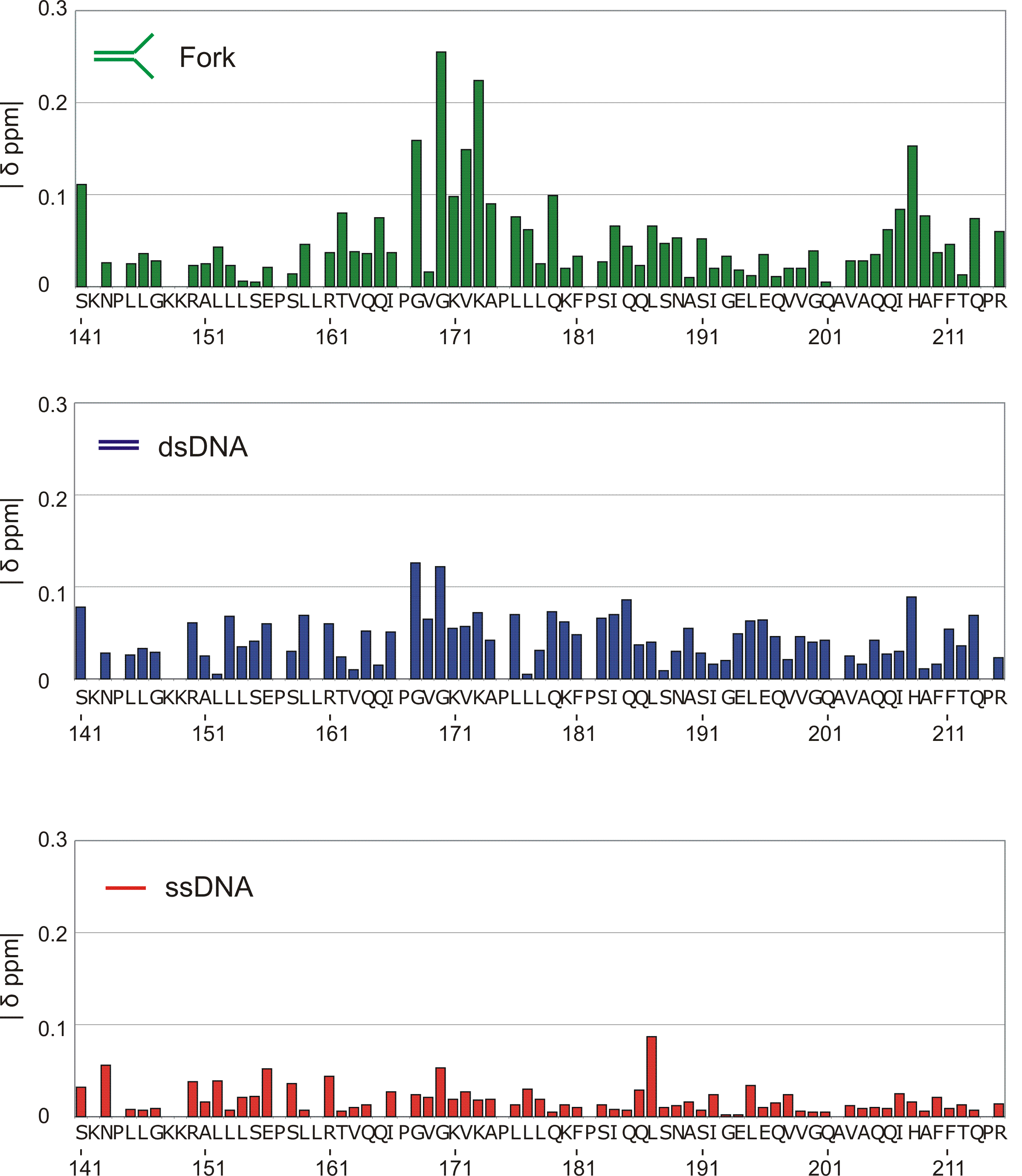


**Supplementary Figure S6.** *Salt dependence of the DNA binding of FAAP24 HhH domain.*

Compound CSP are based on DNA titrations in the presence of 400 μM NaCl using a splayed arm (Fork) substrate with 10 unpaired bases ([400 μM]), a 20bp dsDNA probe ([400 μM]) and a 30nt ssDNA sequence ([200 μM]). The data are presented as compound CSPs for the amide resonances for different DNA substrates at the indicated concentrations.


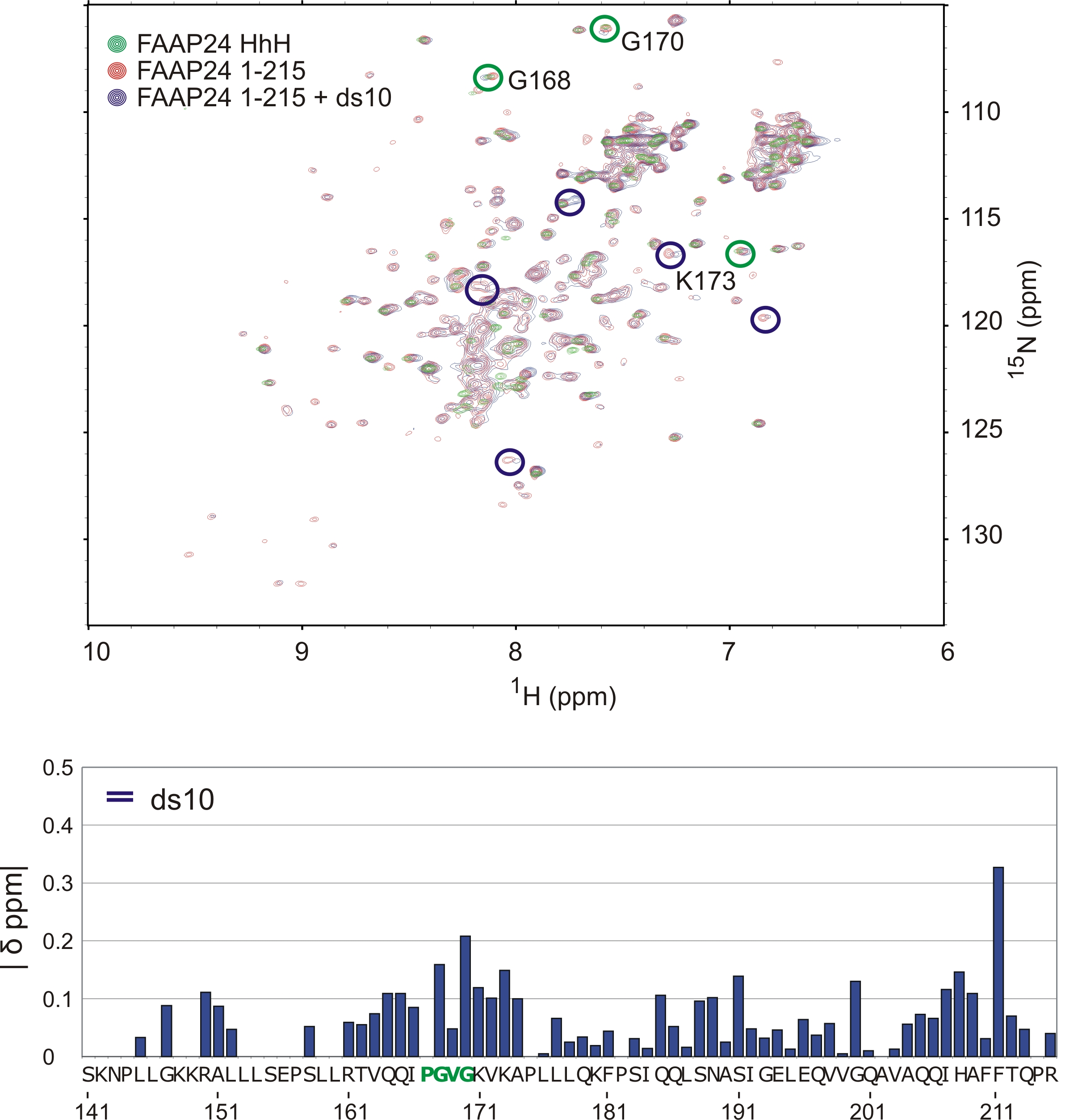


**Supplementary Figure S7.** *dsDNA binding of full length FAAP24.*

[^15^N,^1^H]-HSQC spectra of 200 μM full length FAAP24 (1-215) in the absence (red) or presence (blue) of 200 μM dsDNA (ds10) compared to the HhH domain of FAAP24 (green). The green and blue circles highlight signals that were affected by the addition of dsDNA and that belong to residues within or outside the HhH domain, respectively. The lower panel shows the chemical shift deviation for the HhH domain of FAAP24 (1-215) upon addition of 200 μM 10 bp dsDNA. (residues 1-140 of FAAP24(1-215) are not included since assignments are not available).
